# Identification of promising high-affinity inhibitors of SARS-CoV-2 main protease from African Natural Products Databases by Virtual Screening

**DOI:** 10.21203/rs.3.rs-2673755/v1

**Published:** 2023-03-24

**Authors:** Oudou DIABATE, Cheickna CISSE, Mamadou SANGARE, Opeyemi Soremekun, Segun Fatumo, Jeffrey G. SHAFFER, Seydou DOUMBIA, Mamadou WELE

**Affiliations:** University of Sciences, Technics and Technologies of Bamako (USTTB); University of Sciences, Technics and Technologies of Bamako (USTTB); The African Computational Genomics (TACG) Research group; The African Computational Genomics (TACG) Research group; University of Sciences, Technics and Technologies of Bamako (USTTB); Tulane University School of Public Health and Tropical Medicine; University of Sciences, Technics and Technologies of Bamako (USTTB); University of Sciences, Technics and Technologies of Bamako (USTTB)

**Keywords:** SARS-CoV-2, Main Protease, African Natural Products, Virtual Screening

## Abstract

With the rapid spread of the new severe acute respiratory syndrome coronavirus 2 (SARS-CoV-2), the pathogen agent of COVID-19 pandemic created a serious threat to global public health, requiring the most urgent research for potential therapeutic agents. The availability of genomic data of SARS-CoV-2 and efforts to determine the protein structure of the virus facilitated the identification of potent inhibitors by using structure-based approach and bioinformatics tools.

Many pharmaceuticals have been proposed for the treatment of COVID-19, although their effectiveness has not been assessed yet. However, it is important to find out new-targeted drugs to overcome the resistance concern. Several viral proteins such as proteases, polymerases or structural proteins have been considered as potential therapeutic targets. But the virus target must be essential for host invasion match some drugability criterion.

In this Work, we selected the highly validated pharmacological target main protease M^pro^ and we performed high throughput virtual screening of African Natural Products Databases such as NANPDB, EANPDB, AfroDb, and SANCDB to identify the most potent inhibitors with the best pharmacological properties.

In total, 8753 natural compounds were virtually screened by AutoDock vina against the main protease of SARS-CoV-2. Two hundred and five (205) compounds showed high-affinity scores (less than − 10.0 Kcal/mol), while fifty-eight (58) filtered through Lipinski’s rules showed better affinity than known M^pro^ inhibitors (i.e., ABBV-744, Onalespib, Daunorubicin, Alpha-ketoamide, Perampanel, Carprefen, Celecoxib, Alprazolam, Trovafloxacin, Sarafloxacin, Ethyl biscoumacetate…). Those promising compounds could be considered for further investigations toward the developpement of SARS-CoV-2 drug development.

## 1 Introduction

On January 30th, 2020, the World Health Organization (WHO) has declared the outbreak of the spread of coronavirus (Covid-19) as an international public health emergency [1]. In March 2020 the WHO has classified the COVID-19 as a pandemic [2] showing the rapid spread of the threat due to the Severe Acute Respiratory Syndrome Coronavirus 2 (SARS-CoV-2).

This infection has already infected more than 667,000,000 people and caused more than 6,7 million deaths worldwide (https://coronavirus.jhu.edu/map.html) in January 2023. Although not spared, the Africa continent had more than 258.000 deaths and more than 12,700,000 cases at the beginning of August 2021 (https://www.worldometers.info/ ), and those number are constantly increasing. This rapid spread of the pandemic causes a serious threat to public health, requiring rapid research into therapeutic agents. The development of a new drug requires far too much time and money by conventional methods [3] for situations of extreme urgency such as this; with Omics technologies and innovative Bioinformatics tools, the time and costs are considerably reduced for a better understanding of the mechanisms of action of the involved biomolecules. The availability of viral genomic data and protein structure determination methods has facilitated the identification of potential therapeutic targets and inhibitors using bioinformatics tools.

Thus, many pharmaceuticals have been proposed for the treatment of COVID-19, although their efficiency has not been evaluated yet [4],[5],[6]. Several viral proteins such as proteases, polymerases or structural proteins have been considered as potential therapeutic targets [7].

Natural products (NPs) are broadly defined as chemical substances produced by living organisms. More precised definitions of NPs exist, but they still do not make consensus. Some include all small molecules resulting from metabolic reactions, others classify as “NPs” only the secondary or non-essential metabolism products [8],[9]. In this document, the term Natural Products will designate extracted active compounds from Plants. They have benefited mankind in food, pesticides, cosmetic products, and especially in drugs [10],[8]. The crude extracts from Plants used in traditional medicine contain many pharmacological active compounds [11],[12]. They have shown their healing power in reducing diseases since ancient civilization [13]. These crude medicines can lead to the discovery of other active molecules and eventually to the development of chemical pure drugs that have real beneficial effects. Currently, many prescribed medicines are derived from investigations on natural products. The oldest examples of drugs based on natural products are analgesics, for which willow bark was used to relieve pain, due to salicin, a natural product hydrolyzed into salicylic acid; acetylsalicylic acid, better known as aspirin, is a synthetic derivate used as an analgesic [13]. Some of the notable approved drugs, either from pure or derived NPs, include lefamulin, the aminoglycoside antibiotic plazomicin; tafenoquine succinate, an antimalarial agent; and aplidine, an anticancer agent [14]. These findings are positive proofs that natural products can be used to find efficient drugs.

After the first SARS-CoV epidemic in the early 2000s, the main protease (M^pro^, nsp5) also named chymotrypsin-like protease (3CL^pro^) [15], has been the object of particular attention. Many studies have demonstrated that this protease is a precious therapeutic target due to its essential role in viral replication [16],[17]. Other important coronaviral therapeutic targets include spike protein (S), RNA-dependent RNA polymerase (RdRp, nsp12), NTPase/helicase (nsp13), and papain-like protease (PL^pro^, part of nsp3) [18], [19]. M^pro^ plays a central role in mediating viral replication and transcription functions through extensive proteolytic processing of two replication polyproteins, pp1a (486 kDa) and pp1ab (790 kDa) [20]. It exists only in viruses and is not present in humans. Interestingly, it is the most conserved enzyme among SARS-CoV-2 related viruses [21]. The sequence of the M^pro^ enzyme shows high identity (> 96%) with the SARS-CoV, except for one key residue (Ala285Thr), which may contribute to the high infectivity of the SARS-CoV-2 virus [22],[23]. The functional centrality of M^pro^ in the viral life cycle makes it an interesting target for drug development against SARS and other CoV infections. Therefore, its inhibition may block the production of infectious virus particles and thus alleviate disease symptoms [16],[24]. By capitalizing on this knowledge, M^pro^ is one of the most attractive viral targets for antiviral drug discovery against SARS.

That is why, in this study, we carried out investigations of potential inhibitors of the main protease [25] of SARS-CoV-2 by high-throughput virtual screening of African natural products Databases.

## 2 Materials And Methods

### 2.1 Screening data:

In this study, we used a dataset of compounds from databases of African natural products: AfroDb [26], EANPDB [9], NANPDB [27] and SANCDB [10],[28]. Because these databases did not have the same formats, they were prepared differently. The molecules of the AfroDb and EANPDB databases were prepared using PyRx – Python Prescription 0.8 while those of SANCDB and NANPDB were prepared by Open Babel [29] using homemade scripts. Then all compounds in SDF (Standard Delay Format) format were converted to the pdbqt format. The compounds (Alpha-ketoamide 13b, Daunorubicin, Onalespib, and ABBV-744), have been prepared using AutoDockTools to be used as controls.

### 2.2 Protein preparation:

The main protease (Code PBD: 6Y2F), in complex with alpha-ketoamide 13b (αk-13b) was used as the receptor file. The coordinate file was loaded into PyMol to delete the ligand [6],[30]. Then the protein structure without the ligand was prepared with AutoDockTool-1.5.7 (ADT) [31]. ADT removed non-polar Hydrogens, added the Gasteiger charges, and assigned Solvation parameters and Atom Types. The Computed Atlas of Surface Topography of protein (CASTp) server is an online server that locates and measures pockets and voids on 3D protein structures [32],[33]. It was used to determine the ligand-binding pocket size. Based on the pocket size and taking into account of the alpha-ketoamide 13b position in the crystal structure, the grid box coordinates were set: center_x = −0.000, center_y = −0.704, center_z = −0.000 and size_x = 40, size_y = 40, size_z = 40.

### 2.3 Virtual screening:

Virtual screening is a widely used technique for identifying the top compounds against specific proteins from a library of thousands of compounds [34]. The sum of 8741 molecules was virtually screened with the main protease M^pro^ using AutoDock Vina 1.1.2 [35] in Command Line. After the preparation of the protein and ligands in pdbqt format, all the files were put in the same folder, with a configuration file containing the receptor name, the grid box coordinates, the list of ligands. The Vina script was launched with default parameters.

### 2.4 Lipinski rules of five verifications:

Lipinski’s rule helps to distinguish drug-like from non-drug-like molecules [36]. It states that a drug-like molecule must have at least two of the following rules: Molecular weight less than 500 Dalton, high lipophilicity (expressed as LogP less than 5), less than 5 hydrogen bond donors; less than 10 hydrogen bond acceptors; molar refractivity must be between 40 and 130 [37],[36]. These parameters have been verified using the SWISS ADMET server (http://www.swissadme.ch/) [38] .

### 2.5 Protein-ligand interaction determination:

The interactions between small molecules with the highest affinity scores with M^pro^ was determined using PyMol-2.0 [39] and the Ligplot + v.2.2.4 software [40].

## 3 Results

### 3.1 Natural Compounds from African Databases:

AfroDb is a collection of natural products from African medicinal plants with known bioactivities [26]. It represents the largest diversified collection of 3D structures of natural products covering the entire African continent. These structures can be easily downloaded and used in virtual screening studies (http://african-compounds.org/about/afrodb/). The compounds with a large number of tested biological activities are included in the ZINC database (http://zinc.docking.org/catalogs/afronp/). The South African Natural Compounds Database (SANCDB) is a fully referenced database of natural compounds from sources in South Africa ( https://sancdb.rubi.ru.ac.za/ ) [10]. The Northern African Natural Products Database (NANPDB) is the largest collection of natural compounds produced by indigenous organisms of North Africa (http://african-compounds.org/nanpdb/) [41]. The Eastern Africa Natural Products Database (http://african-compounds.org) containing the structural and bioactivity information of 1870 unique isolated molecules from about 300 source species from the Eastern African region [9]. In total, 8741 natural compounds were obtained from these different databases (Table 1).

### 3.2 SARS-CoV2 active site:

**Figure 1:**
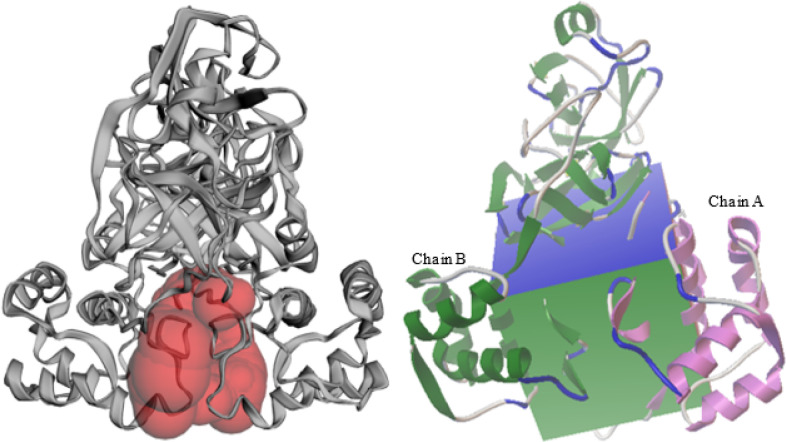
Binding pocket (Red surface) of SARS-CoV-2 M^pro^ determined by CASTp server (left), highlighted in the Grid box, AutoDockTools (right) highlights also the two chains of the protein (chain A in purple and chain B in green).

The structure of SARS-CoV-2 (code PDB: 6Y2F) was used for the virtual screening. The binding pocket determined by the CASTp server was presented in Fig. 1.

The structure was constituted of two protomers (A and B) and a catalytic dyad (His41-Cys145) per protomer, very similar to that of the SARS protease [42]. The enzyme was composed of three domains: the domain I (residues 1–101), domain II (residues 102–184) are mainly made of antiparallel β-sheets, and an α-helical domain III (residues 201–301) [43],[44]. The catalytic domain III contains the Ser284-Thr285-Ile286 segment, an additional domain far from the catalytic dyad. One major difference with SARS-CoV-I is the substitution of the Thr285 residue by Ala [23].

The Fig. 1 left side showed the binding pocket of M^pro^ represented here by the red surface. The molecular surface area of the pocket is 1738.8 Å^3^. This pocket was located between the two protomers. It was used to define a grid box covering the amino acids of the binding site by AutoDockTools (Fig. 1, Right). The grid box volume was made large enough to allow a number of Natural Compounds to dock with the protein.

Indeed, a molecule able to strongly interact between the two protomers could inactivate the enzyme activity, even preventing the protein dimerization [45].

### 3.3 Identification of promising inhibitors from the virtual screening:

In total, 8741 molecules were screened against the SARS-CoV-2 M^pro^ among which two hundred and five (205) molecules have presented affinity scores that varied between − 12.1 Kcal/mol and − 10.0 Kcal/mol.

Among them, fifty-eight (58) were passed through the Lipinski’s rules [37] and twelve (12) did not present any violation of the rule. Those molecules got affinity scores ranging from − 11.2 Kcal/mol and − 10.0 Kcal/mol. Those molecules were considered as promising inhibitors of SARS-CoV-2 (Table 2). The molecules ABBV-744, Daunorubicin and Onalespib described as potent inhibitors of M^pro^ [6] were also screened by using the same docking parameters as controls (Table 2).

The Table 2 showed for each molecule, the parameters of Lipinski’s rules which are molecular weight, logP, number of hydrogen bonds donor and number of hydrogen bonds acceptor. The best twelve molecules identified as SARS-CoV-2 M^pro^ inhibitors did not shown any violation of Lipinski’s rules indicating improved pharmaceutical properties. Moreover, those compounds have shown higher affinity score than standard M^pro^ inhibitors used as controls [6].

### 3.4 SARS-CoV-2 interactions with identified potent inhibitors

For better understanding of the interaction’s details of M^pro^ with the molecules in Table 2, Ligplot and PyMol were used to show interactions (Table 3). The inhibitors showed specific interactions with key residues of M^pro^.

The Table 3 presented the details of the interactions between M^pro^ and the twelve identified compounds. The analysis of the interactions showed that the inhibitors interact mostly with residues Arg4, Leu282, Gly283, Glu288.

**Figure 2:**
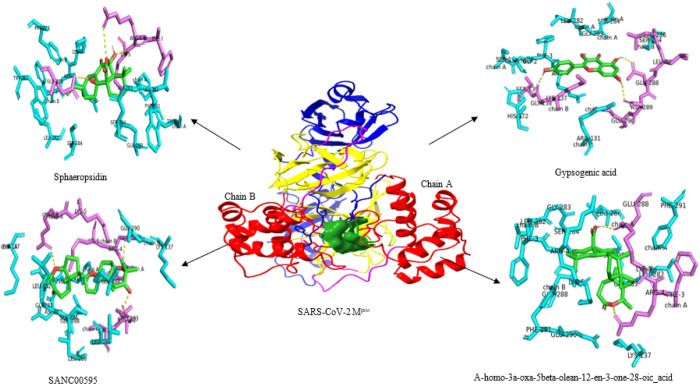
Details of M^pro^ interactions with top four potential inhibitors: - top left, Sphaeropsidin A; - top right, Gypsogenic acid; - bottom left, Yardenone (SANC00595); and - bottom right, A-homo-3a-oxa-5beta-olean-12-en-3- one-28-oic acid. The ligands are illustrated in green stick surrounded by polar residues (magenta) establishing hydrogen bonds(yellow) with the protein and non-polar residues (cyan). M^pro^ is illustrated according to its different domains: domain I in Medium Blue; domain II in Yellow; domain III in red, the catalytic dyad in surface representation (Forest green). A long loop connects domain II to the domain III C-terminal (Magenta).

The Fig. 2 showed the interactions between M^pro^ and the top four inhibitors based on their affinity scores, docked poses and interactions with the protein key amino acids. The details of the other compounds are presented on supplementary data (Fig. 3).

The LigPlot + program was used to map the 2D interactions between M^pro^ and the top four identified compounds (supplementary data; **Fig. 4**).

The analysis of the interaction’s details was summarized in Table 4.

The Table 4 revealed that the main residues of M^pro^ interacting with the ligands were Arg 4, Lys 5 Glu 283, Gly 283 and Glu 288 involving the catalytic domain surrounding the active site dyad (His41-Cys145).

## 4 Discussion

The discovery of new drugs capable of inhibiting the infection caused by SARS-CoV-2 is a global priority in order to put a definitive end to this health emergency. Many studies have demonstrated that the M^pro^ protein was an attractive target against SARS-CoV-2 because of its important role in virus replication, its conservation among other related viruses, and its cleavage specificity different from that of Human proteases [20],[21]. Many crystallographic structures were available for this interesting target which made it very suitable for structure-based drug design [47],[17]. Africa being a rich continent in plant diversity, the first recourse for care is constituted of natural products that are easy to access and less expensive [8]. African natural products have demonstrated antiviral, antifungal and antibacterial properties [46]. These molecules are suitable candidates for high throughput virtual screening against validated drug targets.

In this study, we screened out 8741 compounds from African natural product databases: AfroDb (880), EANPDB (1815), NANPDB (4912) and SANCDB (1134) with the SARS-CoV-2 M^pro^ (code PDB: 6Y2F). The results showed that twelve (12) compounds (Table 2): Sphaeropsidin A, Gypsogenic acid, Yardenone, A-homo-3a-oxa-5beta-olean-12-en-3-one-28-oic acid, Epigallocatechin, NeopellitorinB, Caretroside A, Pallidol, Maslinic acid, Cabralealactone, Tribulus saponin aglycone 3, Olibanumol H and Clionamine D presented high-affinity scores scores ranging from − 11.2 to −10.0 Kcal/mol with SARS-CoV-2 M^pro^. Those molecules presented better affinity scores than standard M^pro^ inhibitors such as 6-Deaminosinefungin (−8.1 Kcal/mol) and UNII-O9H5KY11SV (−8.4 Kcal/mol) proposed by Mohammad and *al*. [4] and Daunorubicin (−9.33 Kcal/mol), Onalespib (−8.21 Kcal/mol), ABBV-744 (−7.79 Kcal/mol) identified by Fakhar and *al*. [6] for the same target in similar screening conditions (Table 2). Moreover, those molecules showed improved pharmacological properties proved by Lipinski’s parameters values. The 12 identified natural products have demonstrated their potential usage as interesting drug precursors of SARS-CoV-2.

The analysis of their interactions with M^pro^ revealed that they bind mainly to protein residues Arg 4, Lys 5 Glu 283, Gly 283 and Glu 288 involving the catalytic domain surrounding the active site dyad (His41-Cys145). Hence the selected compounds could actually prevent the activity of the enzyme. It would be interesting to pursue experimental assays with those promising inhibitors and the SARS-Cov-2 Protein.

Many of these molecules have already shown efficiency in other studies : **Sphaeropsidine A**, against drug-resistant cancer cells [49]; **Gypsogenic acid** against *Bacillus subtilis* and *Bacillus thrungiensis* also as potential antitumor agents [50]; **Yardenone** against hypoxia-inducing factor 1 (HIF-1) activation in breast and prostate tumor cells [51]; **Epigallocatechin** extracted from *Acacia karroo*, was used medicinally to treat diarrhea, colds, dysentery, conjunctivitis and hemorrhages. *Acacia karroo* and other local plant species such as *Artemisia afra, Ziziphus mucronata* and *Eucomis autumnalis*, have been widely used for the treatment of symptoms related to listeriosis [52]; **A-homo-3a-oxa-5beta-olean-12-en-3- one-28-oic Acid**, extracted from *Albizia gummifera*, was used in the indigenous medical system for various nutrients [53].

This study’s findings imply that the inhibitors identified were promising inhibitors of SARS-Cov-2 main protease M^pro^, interesting for the development of efficient drugs against SARS-Cov-2. Further investigations are needed to deepen these findings in the process of drug development.

## 5 Conclusion

In this study, we identified twelve (12) compounds (Sphaeropsidin A, Gypsogenic acid, Yardenone, A-homo-3a-oxa-5beta-olean-12-en-3- one-28-oic acid, Epigallocatechin, Neopellitorine B, Caretroside A, Pallidol, Maslinic acid, Cabralealactone, Tribulus saponin aglycone 3, Olibanumol H, Clionamine D) from African Natural Product which showed high-affinity and validated interesting pharmacological properties against the main protease M^pro^, the most attractive target of SARS-CoV-2. Further investigations have to be pursued with the identified compounds to foster the way into the development of new drugs against the COVID-19 virus.

## Figures and Tables

**Table 1:** Natural Compounds from African databases used for virtual screening

Database	SANCDB	NANPDB	EANDPB	AfroDb	Total
Number of compounds	1134	4912	1815	880	8741
References	R. Hatherley et al.,2015 [10]	Fidele Ntie-Kang et al., 2017 [27]	Conrad V. Simoben et al., 2020 [9]	Ntie-Kang et al., 2013 [26]	

**Table 2:** List of the most promising potent inhibitors of SARS-CoV-2 M^pro^ from African Natural Compounds with improved drug-like properties and standard inhibitors

No	Compounds	Molecular weight (Dalton)	Log(P)	Hydrogen donor	Hydrogen acceptor	Affinity (Kcal/mol)
1	**Sphaeropsidin A (NA)**	346.42	2.27	2	5	−11.2
2	**Gypsogenic acid (NA)**	486.68	2.98	3	5	−10.6
3	**Yardenone (**SANC00595**)**	488.7	4.51	0	5	−10.5
4	**A-homo-3a-oxa-5beta-olean-12-en-3- one-28-oic acid (EA)**	470.691	−3.503	1	5	−10.4
5	**Epigallocatechin (NA)**	306.27	0.42	6	7	−10.4
6	**Neopellitorine B (NA)**	235.37	3.60	0	1	−10.4
7	**Caretroside A (NA)**	402.48	2.20	3	6	−10.3
8	**Pallidol (NA)**	454.47	2.51	5	6	−10.3
9	**Maslinic acid (NA)**	472.70	3.38	3	4	−10.2
10	**Cabralealactone (NA)**	414.62	3.86	0	3	−10.2
11	**Olibanumol H (NA)**	460.73	4.30	3	3	−10.1
12	**Clionamine D (**SANC00290**)**	401.54	2.43	2	5	−10.0
Known M^pro^ Inhibitors used as controls						
1	**Alpha-ketoamide 13b**	593.7	3.96	4	7	−9.2
2	**Daunorubicin**	527.5	2.66	5	11	−9.1
3	**Onalespib**	409.5	3.84	2	5	−8.4
4	**ABBV-744**	491.6	3.65	3	5	−8.0

NA = NANPDB; EA = EANPDB and SANC00290, SANC00595 = compounds id in SANCDB.

**Table 3:** Interactions between with M ^pro^ and the thirteen potent inhibitors.

Molecules	Interactions with Protein via	
	Chain A	Chain B
Sphaeropsidin A	Arg-4, Lys-5	Glu-288
Gypsogenic acid	Arg-4	Glu-283
Yardenone	Arg-4, Gly-283	Arg-4
A-homo-3a-oxa-5beta-olean-12-en-3- one-28-oic acid	None	Arg-4, Glu-288
Epigallocatechin	Lys-137, 2Glu-288, Asp-289, Glu-290	Arg-4
Neopellitorine B	Lys-137, Thr-169, Leu-287, Asp-289	Thr-280, 2Glu-283
Caretroside A	Glu-288	Trp-207, Gly-283, Lys-5
Pallidol	Phe-3, Asp-289	Lys-5, Glu-288
Maslinic acid	Phe-3	Leu-282
Cabralealactone	Arg-4	Leu-282
Olibanumol H	Arg-4, Gly-138	Lys-5, 2Phe-3
Clionamine D	Phe-3, Leu-282	Phe-3, Leu-282

**Table 4:** Summary of interactions between M^pro^ and the top four compounds:

	Number of H Bonds	M^pro^ Residues involved in	
		H Bonds	VdW interactions
Sphaeropsidin A	3	Arg-4, Lys-5 (Chain A) Glu-288 (Chain B)	Phe-291, Phe-3, Ser-284, Glu-288, Leu-282, (Chain A) Lys-5, Trp-207, Phe-291 (Chain B)
Gypsogenic acid	2	Arg-4 (chain A) Glu-283 (chain B)	Gly-283, Phe-3, Gly-2, Leu-282 (Chain A) Leu-286, Lys-137 (Chain B)
Yardenone	3	Arg-4, Gly-283 (Chain A) Arg-4 (chain B)	Lys-5, Leu-282, Glu-290, Glu-288, Gly-283 (Chain A) Glu-290, Gly-283, Leu-282, Lys-5, Lys-137, Glu-288 (Chain B)
A-homo-3a-oxa-5beta-olean-12-en-3-one-28-oic acid	2	Arg-4, Glu-288 (chain B)	Leu-282, Gly-283, Arg-4, Phe-3, Phe-291, Lys-5 (Chain A) Phe-291, Phe-3, Lys-5, Glu-288 (Chain B)

## Data Availability

The databases used during the current study were available in http://african-compounds.org/about/afrodb/ for AfroDB; https://sancdb.rubi.ru.ac.za/ for SANCDB; http://african-compounds.org/nanpdb/ for NANPDB and EANPDB.
